# Serum Sickness-Like Reactions After Pneumococcal Vaccination

**DOI:** 10.7759/cureus.17877

**Published:** 2021-09-10

**Authors:** Bing-Syuan Chung, Wei-Ting Liu, Po-Wei Chen

**Affiliations:** 1 Department of Internal Medicine, National Cheng Kung University Hospital, College of Medicine, National Cheng Kung University, Tainan, TWN; 2 Department of Dermatology, National Cheng Kung University Hospital, College of Medicine, National Cheng Kung University, Tainan, TWN

**Keywords:** serum sickness-like reactions, serum sickness, pneumococcal vaccine, side effects, arthritis

## Abstract

Serum sickness or serum sickness-like reactions (SSLRs) constitute a rare complication that manifests in individuals after receiving vaccinations. In this case report, we present a patient with typical symptoms of SSLRs after pneumococcal vaccination. The time course of this disease and our diagnostic process are documented in detail. The literature related to serum sickness and SSLRs is also reviewed.

## Introduction

Pneumococcal vaccination protects against *Streptococcus pneumoniae (S. pneumoniae)*, which is the most common pathogen causing community-acquired pneumonia in Taiwan. Pneumococcal vaccination is very common since it is part of the immunization schedule in children and is also indicated in elderly people and adults with health conditions that put them at increased risk of pneumococcal infection or its complications. Serum sickness is an immune response caused by type III hypersensitivity reaction [1], which is associated with heterologous serum exposure to agents such as the rabies vaccine, antivenom, and murine monoclonal antibodies [2-4]. Serum sickness-like reactions (SSLRs), the pathophysiology of which is not fully understood, are reactions to drugs, infections, and vaccines that cause symptoms similar to serum sickness. Vaccine-induced SSLRs are rare, and only a few cases of pneumococcal vaccine-induced SSLRs have been reported [5,6]. In this report, we present the first case of SSLRs after pneumococcal vaccination in an immunocompetent adult.

## Case presentation

A 63-year-old man with type II diabetes, hypertension, and dyslipidemia presented to our emergency department with sudden-onset left inguinal pain for one day. Nine days earlier, he had received, for the first time in his life, a 13-valent pneumococcal conjugate vaccine (Prevnar 13; Pfizer Inc., Brooklyn, NY). Rashes had developed bilaterally on the lower limbs one week later, which had gradually extended to the trunk region the next day. No skin rash had been identified on the injection site (middle of his left deltoid muscle). The patient did not have an allergy history to any kind of food or medication. On the ninth day, he had developed left inguinal pain, which prevented him from walking. Thus, he had been referred to our hospital.

**Figure 1:**
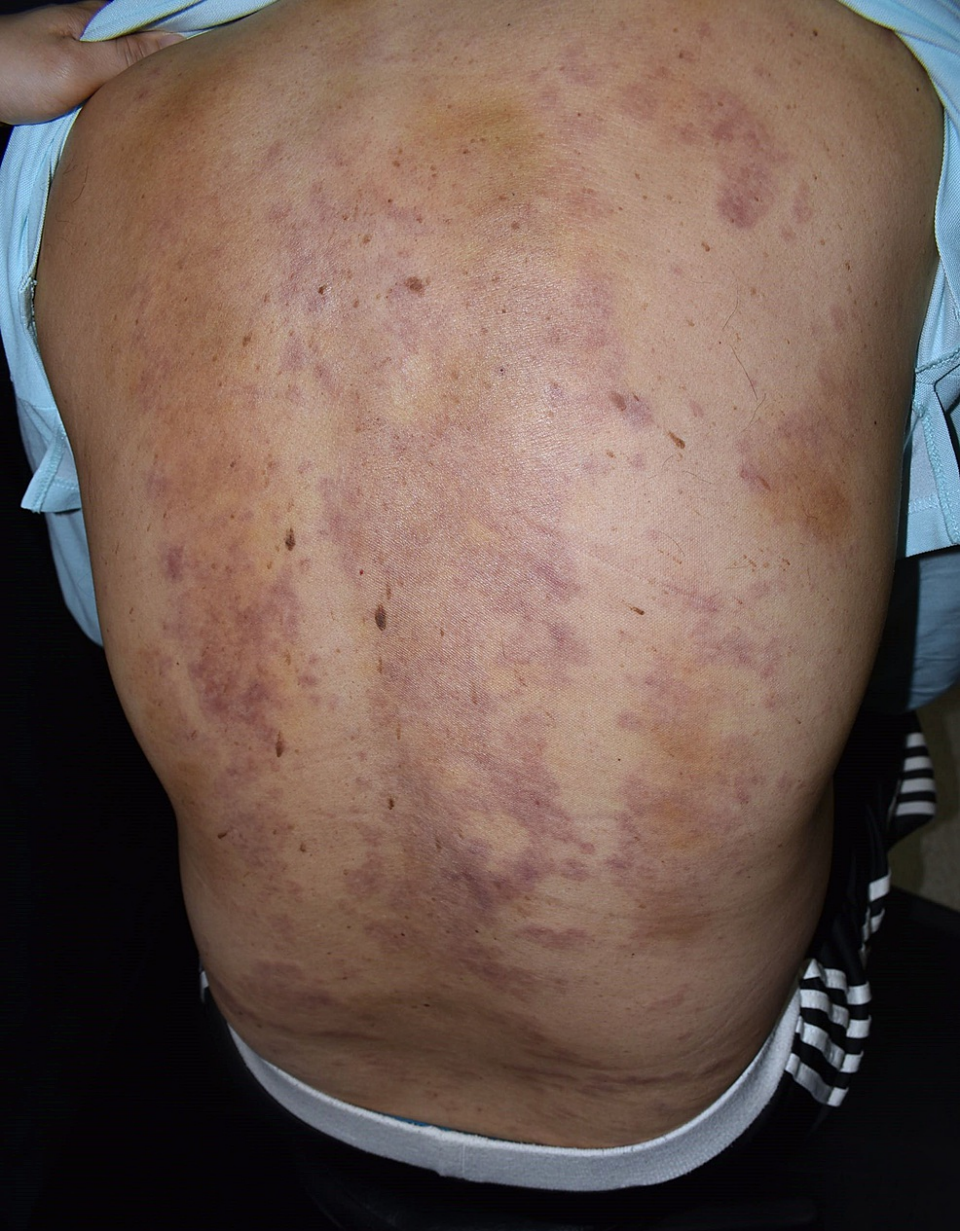
Rashes over the back On day nine after the vaccination, widespread erythematous urticarial plaques in geographic patterns with scattered light-brown patches were observed. Some rashes had an annular or targetoid appearance with a flat, violaceous center and a raised, wheel-like border, especially over the lower back

**Figure 2:**
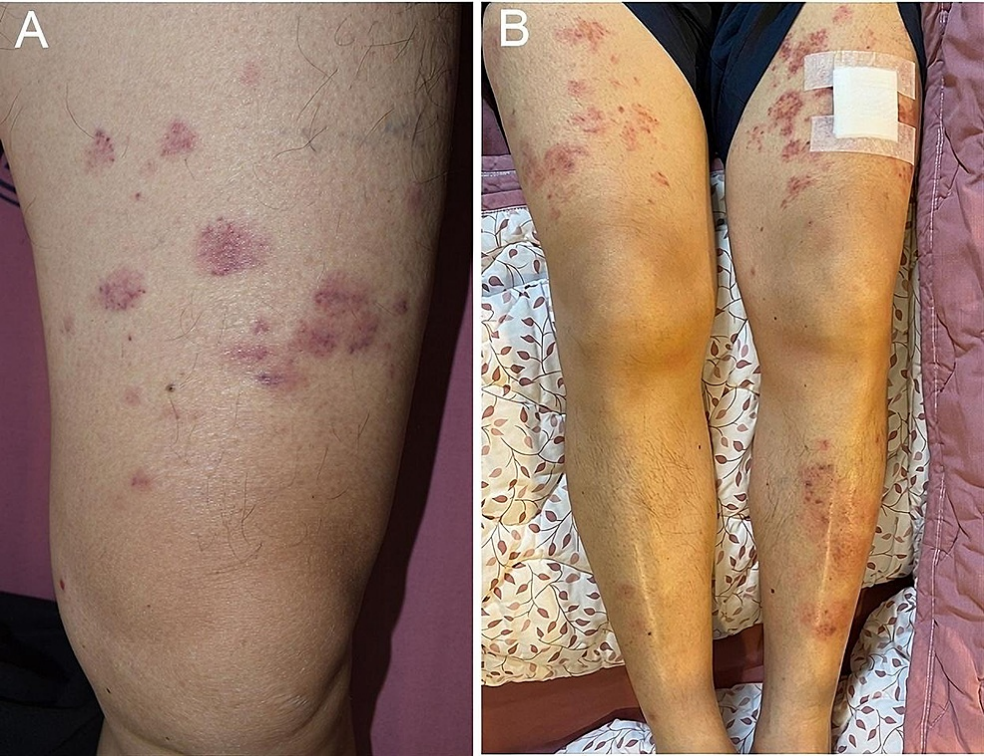
Rashes over thighs and legs A. On day nine after vaccination, rashes over the thighs consisting of several oval-shaped erythematous patches centrally with some pinpoint-sized purpuric macules were noted. B. On day 12 after vaccination, some rashes expanded centrifugally with an annular configuration and even more purpuric change over the bilateral thigh and leg

On examination, his body temperature was 38.2 °C, pulse rate was 100 beats per minute, respiratory rate was 15 breaths per minute, and blood pressure was 126/53 mmHg. His left hip revealed no swelling or local heat; however, pain upon passive or active movement in the left hip joint was noted. His back was extensively covered with erythematous urticarial plaques (Figure 1), and his bilateral thighs were covered with erythematous patches (Figure 2). None of the skin lesions were itchy or painful. No lymphadenopathies were noted.

His white blood cell (WBC) count was 14.8 x 10^3^/uL, including 59% neutrophils, 5% monocytes, 20% lymphocytes, and 15% atypical lymphocytes. Electrolyte levels, renal function, and liver function were normal. Urinary analysis revealed no pyuria, bacteriuria, hematuria, but mild proteinuria (30 mg/dL) was found. His C-reactive protein level was 126.8 mg/L, lactate dehydrogenase level was 232 U/L, and ferritin level was 862 ng/mL. Moreover, his rheumatoid factor, anti-cyclic citrullinated peptide antibody, antinuclear antibody, antineutrophil cytoplasmic antibody, C3, C4, serum electrophoresis, and serum immunofixation were normal. The IgG level increased to 2,740 mg/dL, but the IgA and IgM levels were normal.

**Figure 3:**
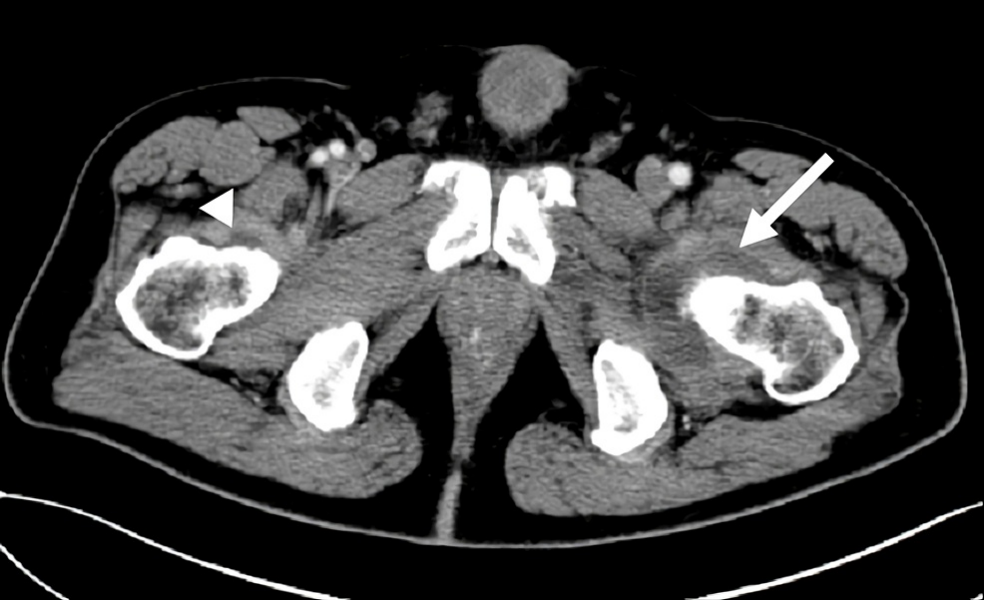
CT image of left hip arthritis On day nine after vaccination, left hip joint arthritis with fluid accumulation and increased synovial enhancement (arrow) were observed; minimal fluid deposition in the right hip joint (arrowhead) could also be seen CT: computed tomography

Abdominal CT with contrast enhancement revealed fluid deposition around the left hip with increased synovial enhancement. Minimal fluid deposition in the right hip joint was noted as well (Figure 3). Neither hepatomegaly nor splenomegaly was detected. We performed arthrocentesis in his left hip joint owing to suspected arthritis. The synovial fluid aspirated from his left hip joint was yellow in color and turbid with normal viscosity. The synovial leukocyte count was 47,681/mm^3^, including 42% neutrophils, 37% macrophages/monocytes, 17% lymphocytes, and 4% atypical lymphocytes. Skin biopsies on the back and thighs were also performed.

**Figure 4:**
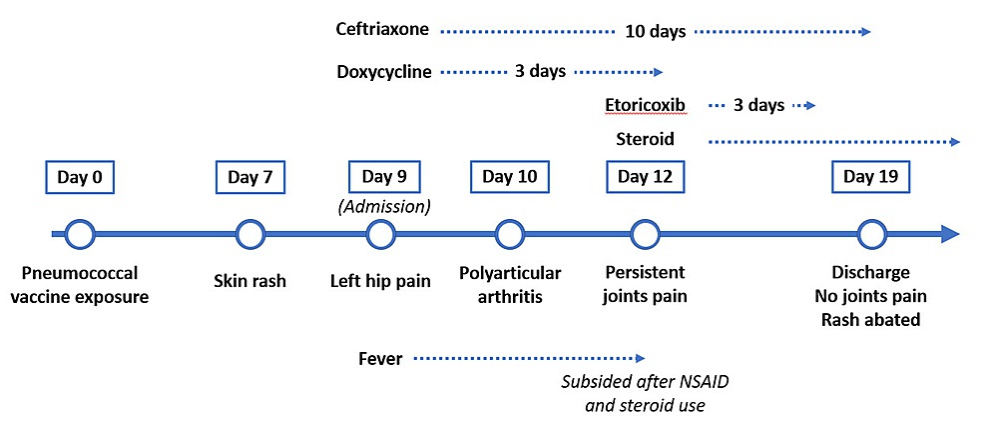
Time course of SSLRs in the patient Dosage of ceftriaxone: 2 g/day; dosage of doxycycline: 200 mg/day; dosage of etoricoxib: 90 mg/day; dosage of steroid: methylprednisolone 40 mg/day during days 12-15, prednisolone 20 mg/day during days 16-23, 10 mg/day after day 24 until outpatient follow-up one month later SSLRs: serum sickness-like reactions; NSAIDs: nonsteroidal anti-inflammatory drugs

**Figure 5:**
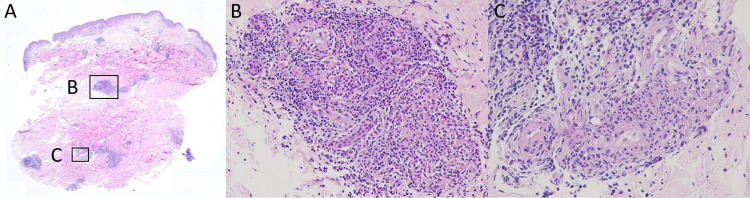
Skin biopsy specimen The skin biopsy taken from one erythematous to violaceous papule over the back A. In the scanning view, there are superficial and deep perivascular and periadnexal dense inflammatory cell infiltrates. B. A dense, mixed lymphocytic and neutrophilic infiltrate surrounding eccrine glands associated with necrosis of a few epithelial cells of the secretory coils (200x). C. At the dermal subcutaneous junction, there is a moderately dense perivascular inflammatory infiltrate with nuclear dust and fibrin deposit in a few medium-sized blood vessels (400x)

Empirical antibiotics, including ceftriaxone and doxycycline, were administered. However, the patient developed aggravated joint pain, including in the bilateral shoulder, hip, and knee joints, along with persistent fever on day two of admission. Joint sonography revealed fluid deposition in all aforementioned joints. Left knee arthrocentesis was performed, and the results were similar to those of the synovial fluid aspirated from the left hip joint but with higher leukocyte counts (106,795/mm^3^, including 56% neutrophils, 19% macrophages/monocytes, and 25% lymphocytes). On day three of admission, a high fever of approximately 39 °C and aggravated joint pain were noted. Nonsteroidal anti-inflammatory drugs (NSAIDs) and steroids were initiated, and the fever subsided dramatically on the same day. The joint pain and skin rashes also resolved to a considerable extent in the following few days. The timeline of the patient’s clinical course is illustrated in Figure 4. Subsequent culture reports, including bacterial, viral, fungal, and tuberculosis tests from blood and synovial fluid, were negative, as were HIV and *Neisseria gonorrhoeae* nucleic acid tests. Skin pathology on the thighs and back revealed superficial and deep, perivascular, and periadnexal neutrophilic dermatitis, which were compatible with SSLRs. There were also features of medium-sized vasculitis (Figure 5).

On day 10 of admission, the joint pain had almost subsided completely, and joint sonography revealed only minimal fluid accumulation. The patient was discharged with low doses of oral NSAIDs and steroids. On follow-up at one month, no sequelae were noted.

## Discussion

Our patient, a 63-year-old man, presented with left hip arthritis that progressed to polyarthritis in the large joints, including bilateral shoulder, hip, and knee joints. Symptoms were concurrent with skin rashes on the bilateral legs and trunk and systemic inflammatory response syndrome. Initial differential diagnoses were polyarticular septic arthritis; autoimmune systemic diseases such as adult-onset Still’s disease, rheumatoid arthritis, and systemic lupus erythematosus; and crystal arthritis. His synovial WBC counts were high, especially the second aspiration in the left knee joint (106,795/mm^3^), but the concentration of neutrophils was only 40-50%. Apart from bacterial infection, we also considered viral, tuberculosis, and fungal infections. However, polyarticular septic arthritis rarely occurs in immunocompetent adults. His HbA1c level was lower than 7, and he had never received any immunosuppressants. He and his family did not have any history of congenital immunodeficiency or opportunistic infections. Symptoms of both polyarthritis and fever did not abate after empirical administration of antibiotics, and subsequent culture reports did not reveal the presence of any pathogen. Thus, the diagnosis of polyarticular septic arthritis was less likely. The patient did not have any history of autoimmune diseases, and autoimmune titers revealed negative findings. Yamaguchi’s criteria for adult-onset Still’s disease were not fulfilled. Crystal arthritis was also ruled out after microscopic examination of synovial fluid. Differential diagnoses of cutaneous urticarial eruption with arthritis were autoimmune skin eruption, infection-related skin eruption, vaccination-related skin rash, or adult-onset Still’s disease. Because subsequent skin biopsy reports were consistent with SSLRs, serum sickness or SSLRs were highly suspected. Considering his history of vaccine exposure one week earlier and his C3 and C4 levels being normal, the diagnosis of pneumococcal vaccine-induced SSLRs was finally made.

Diagnosing serum sickness or SSLRs is challenging because it is based on exclusion, symptoms, and the temporal relationship between the onset of symptoms and the exposure to triggers. The term “serum sickness” was first described by von Pirquet and Schick, who illustrated the side effects in patients who had received horse serum for treating diphtheria and scarlet fever in the 18th century [7]. Serum sickness causes immunocomplex deposition in blood vessels and tissues, which activates the classical complement pathway, and then induces tissue damage. Serum complement levels of C3 and C4 would usually decrease [1]. The typical symptoms of serum sickness are fever, rashes, and joint pain. Symptoms usually develop one to two weeks after the exposure to triggers but may start earlier if such exposure is not the first of its kind. Symptoms may last from weeks to months, and patients usually respond well to antihistamines or NSAIDs. In severe cases, steroid administration may be necessary. In rare and extremely severe cases, plasmapheresis can be used to shorten the time to recovery. In some cases where the triggers cannot be discontinued, plasmapheresis might prevent serum sickness [8,9]. Serum sickness is generally a benign disease and is mostly self-limiting.

Compared with serum sickness, SSLRs do not form immune complexes, and patients have normal C3 and C4 levels because the complement system is not activated [10]. SSLRs usually develop after the administration of antibiotics such as cefaclor, penicillin, metronidazole, ciprofloxacin, and clarithromycin [11-15]. Vaccine-triggered SSLRs are rare, but those induced by rabies, influenza, and pneumococcal vaccines have been reported [5,6,16,17]. However, SSLRs induced by pneumococcal vaccines are still relatively rare compared with those induced by rabies and influenza vaccines, and most cases of SSLRs induced by vaccines are found in children. The treatment protocol for SSLRs is the same as that for serum sickness.

In Taiwan, two types of pneumococcal vaccines are available, namely the pneumococcal conjugate vaccine (PCV13 or Prevnar 13) and pneumococcal polysaccharide vaccine (PPSV23 or Pneumovax 23, Merck & Co., Inc., Kenilworth, NJ). Prevnar 13, which was used in our patient, contains the capsular polysaccharide of 13 serotypes of *S. pneumoniae* conjugated to a nontoxic mutant of the diphtheria toxin called CRM 197 to boost immune responses. Common side effects of Prevnar 13 include local reactions, fever, fatigue, rashes, muscle pain, and diarrhea. Most local reactions and systemic side effects are mild or moderate in severity. A previous study has reported that severe adverse events within one month after vaccine injection were rare (7%) and not statistically significant compared with the placebo group [18]. In our literature review, we observed that pneumococcal vaccine-induced SSLRs mostly occurred in children and in only one HIV-infected adult [5,6]. Thus, our case is the first one that documents an immunocompetent adult with SSLRs after receiving pneumococcal vaccination.

## Conclusions

SSLRs usually develop one to two weeks after the first exposure to triggers. Patients presenting with fever, skin rashes, and arthritis should be suspected of having serum sickness or SSLRs, especially if the temporal relationship between the onset of symptoms and the exposure to triggers is consistent. Although pneumococcal vaccine-induced SSLRs are very rare, they may still develop in children and immunocompetent adults.
